# EpiMCI: Predicting Multi-Way Chromatin Interactions from Epigenomic Signals

**DOI:** 10.3390/biology12091203

**Published:** 2023-09-03

**Authors:** Jinsheng Xu, Ping Zhang, Weicheng Sun, Junying Zhang, Wenxue Zhang, Chunhui Hou, Li Li

**Affiliations:** 1 Hubei Key Laboratory of Agricultural Bioinformatics, College of Informatics, Huazhong Agricultural University, Wuhan 430070, China; 2 Food Science Program, Division of Food, Nutrition and Exercise Sciences, University of Missouri, 1406 E Rollins Street, Columbia, MO 65211, USA; 3 China State Key Laboratory of Genetic Resources and Evolution, Kunming Institute of Zoology, Chinese Academy of Sciences, Kunming 650223, China; 4 Hubei Hongshan Laboratory, Huazhong Agricultural University, Wuhan 430074, China

**Keywords:** hypergraph neural network, multi-way chromatin interaction, HiPore-C, histone modification, chromatin compartment

## Abstract

**Simple Summary:**

Dissecting the relationship between epigenome signals and three-dimensional multi-way chromatin interactions remains a challenging problem. The emergence of high-throughput Pore-C technology offers promising hope for tackling this issue. In this study, we proposed the EpiMCI, a framework based on a hypergraph neural network, aiming to reconstruct multi-way chromatin interactions from epigenomic signals. The model obtained AUCs of 0.981 and 0.984 using the GM12878 and K562 datasets, outperforming the existing methods. The EpiMCI can be used to denoise multi-way contact sequencing data and improve data quality. The embeddings obtained from the EpiMCI reflect the exact genome structure, confirming the rationality of the EpiMCI from a biological perspective. Thus, the EpiMCI is a promising framework for reconstructing multi-way chromatin interactions from epigenomic signals and can be applied to studies related to multi-way chromatin interactions reconstruction.

**Abstract:**

The recently emerging high-throughput Pore-C (HiPore-C) can identify whole-genome high-order chromatin multi-way interactions with an ultra-high output, contributing to deciphering three-dimensional (3D) genome organization. However, it also brings new challenges to relevant data analysis. To alleviate this problem, we proposed the EpiMCI, a model for multi-way chromatin interaction prediction based on a hypergraph neural network with epigenomic signals as the input. The EpiMCI integrated separate hyperedge representations with coupling hyperedge information and obtained AUCs of 0.981 and 0.984 in the GM12878 and K562 datasets, respectively, which outperformed the current available method. Moreover, the EpiMCI can be applied to denoise the HiPore-C data and improve the data quality efficiently. Furthermore, the vertex embeddings extracted from the EpiMCI reflected the global chromatin architecture accurately. The principal component analysis suggested that it was well aligned with the activities of genomic regions at the chromatin compartment level. Taken together, the EpiMCI can accurately predict multi-way chromatin interactions and can be applied to studies relying on chromatin architecture.

## 1. Introduction

In eukaryotes, genomes are folded into three-dimensional (3D) structures that facilitate gene regulation within the nucleus [1,2,3,4]. Current chromatin conformation capture technologies, such as Hi-C and ChIA-PET [5,6,7], have enabled the genome-wide characterization of chromatin organization, including A/B compartments [6], topologically associating domains (TADs) [8,9,10] and chromatin loops [11,12,13]. However, the assays mentioned above can only capture interactions between genome loci that are in close position to ligate directly. These interactions were captured and sequenced pairwise, losing the information from multiple chromatin loci (≥3) that interacted simultaneously in the same nucleus [14].

Recently, new burgeoning technologies, including GAM [15], SPRITE [16], Tri-C [17], multi-contact 4C [18], ChIA-Drop [19], Pore-C [20] and HiPore-C [21], have been developed to study high-order 3D interactions among multiple genomic loci within individual nuclei. Beagrie et al. identified an abundance of three-way contacts and revealed regulatory elements that form higher-order contacts using GAM [15]. Using SPRITE, Quinodoz et al. found two major inter-chromosomal hubs around nuclear bodies [16]. Based on ChIA-Drop, Zheng et al. detected multi-way interactions mediated by RNAPII and characterized potential promoter-centered multivalent interactions. Thus, they, proposed a promoter-centered, one-sided extrusion model for RNAPII-mediated transcription [19]. In addition, Deshpande et al. identified synergies, which were genome loci sets with a frequency significantly higher than the background and revealed an enrichment of the multi-way contacts in the enhancers and promoters in active chromatin and highly transcribed genes via Pore-C [20]. Zhong et al. revealed a relationship between allele-specific topology and 3D genome structures using HiPore-C, an optimized protocol based on Pore-C [21]. Taken together, these data depicted the accessibility of higher-order interactions in 3D genomics exploration, which was unachievable by pairwise interactions captured directly using conventional experiments.

Epigenomic modifications and 3D genomic interactions are tightly associated. However, they are currently measured by distinct technologies and an integrative interpretation is still lacking [22,23]. Various computational methods based on epigenomic signals have been developed to predict chromatin interactions [4,23,24,25,26,27]. Cao et al. proposed a method called the joint effect of multiple enhancers (JEME) for predicting the target genes of the enhancers in specific samples [24]. The method used histone marks as the features and used random forest to capture the non-linear relationships between these features and predict potential enhancer targets. He et al. introduced IM-PET, an integrated method for predicting enhancer targets [4]. It developed multiple features based on omics data and integrated them probabilistically to make robust predictions of enhancer–promoter pairs. Roy et al. developed a computational approach named the Regulatory Interaction Prediction for Promoters and Long-range Enhancers (RIPPLE) for predicting enhancer–promoter interactions [25]. By adopting a random forest approach and a regularized regression method, RIPPLE could be applied to predict interactions in a new cell line and generate genome-wide interaction maps. Singh et al. proposed a deep learning model called SPEID to predict enhancer–promoter interactions based on sequence-based features [26]. Whalen et al. presented TargetFinder, a computational method to predict individual enhancer–promoter interactions based on epigenomic signals [27]. Pierro et al. proposed an ensemble method, which used epigenetic marks as the input and followed an energy landscape model for chromatin organization to generate 3D chromosome conformations [28]. Liu et al. presented DeepChIA-PET, a supervised deep learning approach to predict ChIA-PET interactions using Hi-C and epigenomic signals [29]. Yang et al. designed Epiphany, a neural network to predict cell-type-specific Hi-C contact maps from widely available epigenomic tracks [30]. The model exploited bidirectional long short-term memory layers and a generative adversarial network architecture to generate the predicted contact map.

Although the relation of chromatin pairwise interactions and the epigenomic marks has been explored, the underlying multi-way contacts information has not been successfully decoded. The methods mentioned above cannot describe contacts involving more than two genomic loci, which results in a loss of higher-order structural information [31,32]. Hypergraph, a generalization of graph theory, was proposed to resolve these issues [33]. Hypergraph is widely used in email communication networks, co-authorship networks, film actor/actress networks and protein–protein interaction networks [34]. It can capture higher-order connectivity patterns and represent multi-dimensional relationships unambiguously in genomic networks, filling the gap that image-based and graph-based methods could not reach [35]. If an interaction consists of more than two nodes, it can be represented as a hyperedge. The graph containing multiple hyperedges is then called as a hypergraph. Indeed, multi-way chromatin interactions in the whole genome construct a hypergraph and each multi-way interaction corresponds to a hyperedge within the hypergraph [36,37]. To our knowledge, only one study probed multi-way chromatin interactions using a hypergraph neural network. The study proposed MATCHA, which considered only the structural information of the original data for prediction [37]. Inspired by the idea, we developed the EpiMCI (Epigenomics-based Multi-way Chromatin Interactions prediction), a model based on a hypergraph neural network, to derive 3D multi-way chromatin interactions from epigenomic data. The EpiMCI uses HiPore-C multi-way chromatin interactions data and epigenomic signals as the input and then extracts patterns from the corresponding hypergraph. By combing the separating hyperedge representations and the coupling hyperedge information, the EpiMCI can accurately predict the probability for a group of bins with a simultaneous interaction. The results revealed that the EpiMCI can denoise HiPore-C data efficiently. The embeddings of the genome loci from the model also reflected their 3D spatial positioning in terms of the chromatin compartments. Collectively, the EpiMCI provides a bridge connecting the 1D epigenomic information and 3D multi-way interactions of the genome. It also has great potential to be applied in multi-way chromatin interaction exploration where only epigenomic information is available and can provide new insights into nuclear genome structure and function.

## 2. Materials and Methods

### 2.1. Data Source

The GM12878 and K562 HiPore-C data were downloaded from the NCBI GEO repository using the access number GSE202539. The GM12878 hg38-based in-situ Hi-C pair data were downloaded from ENCODE using the access number ENCFF447ERX. The K562 hg38-based in-situ Hi-C were downloaded from the 4DN data portal using the access number 4DNFI2R1W3YW (https://data.4dnucleome.org/experiment-set-replicates/4DNESI7DEJTM/ accessed on 14 March 2023). The epigenomic signal, including the histone modification ChIP-seq, transcription factor ChIP-seq and ATAC-seq data, were downloaded from the ENCODE database and are listed in Table S3.

### 2.2. Data Preprocessing

The processed HiPore-C fragments were converted into a cluster file, i.e., a read followed by several fragments belonging to this read, using our custom python script. The multi-way interaction of the HiPore-C reads were also decomposed into pairwise contacts to creates a medium-format contact matrix, which was further normalized by KR matrix balancing. The in-situ Hi-C pairs data were converted into ‘.hic and .cool’ files using juicer and cooler (https://github.com/open2c/cooler accessed on 14 March 2023) [38]. The epigenomic signals were defined as peak numbers within the 1 Mb bin. The reproducibility score between the two Hi-C datasets was calculated using GenomeDISCO (https://github.com/kundajelab/genomedisco accessed on 14 March 2023), which compared the contact maps of the 3D genome structures [39].

### 2.3. Hypergraph Definition and Prediction Problem Statement

A hypergraph is a generalization of a graph [33]. This data structure enabled us to model multi-way relations, where the edges could be incident to more than two nodes. Mathematically, it is defined as H=(V, E, W, U), where $V=\{v_{1},\;v_{2},\ldots ,v_{N}\}$ and |V|=N represents the vertex sets in the hypergraph; $E\;=\{e_{1},\;e_{2},...,\;e_{M}\}$ and |E|=M represents the sets of hyperedges ($e_{i}=(v_{1}^{(i)},{\;v}_{2}^{\left(i\right)},\ldots ,{\;v}_{k}^{\left(i\right)})$, k = 1, 2,…, N); $W\in \;R^{M\times M}$ represents the diagonal matrix of the hyperedge weights and $U\in \;R^{N\times N}$ represents the diagonal matrix of the vertex weights. Each hyperedge connects more than two vertices, i.e., |*e*| > two, which indicates the degree of the corresponding hyperedge. If all the hyperedges in a hypergraph have the same number of nodes $\left|e_{i}\right|=k,\;\forall e_{i}\in E$, it is referred to as a *k*-uniform hypergraph. Otherwise, it is referred to as a non-uniform hypergraph. It is worth noting that the multi-way chromatin interactions used in this study contained different numbers of fragments from three to six (described in Section 3.1). Each interaction was then regarded as one hyperedge and the constructed hypergraph was a non-uniform hypergraph.

The hypergraph structure has Its own exclusive representation and generally is denoted by an incidence matrix $H\in {\left\{0,\;1\right\}}^{N\times M}$*,* with each entry H (v, e) indicating whether the vertex v is contained in the hyperedge e.

(1) $$H(v,\;e)=\left\{\begin{array}{l} =1,\;\;\;\;\;\;\;\;\mathrm{if}\;v\in e\\ =0,\;\;\;\;\;\;\;\;\mathrm{others} \end{array}\right.\;\;$$

In this form, the vertex degree can be defined as a diagonal matrix $D_{v}\;\in \;R^{N\times N}$

(2) $$D_{ii}=\sum_{e=1}^{M}W_{ee}H_{ie}$$

and the hyperedge degree can be defined as a diagonal matrix $D_{e}\;\in R^{M\times M}$.

(3) $$D_{ee}=\sum_{i=1}^{N}H_{ie}$$

We regard the problem as a hyperedge binary classification task, i.e., given a group of vertices $\left\{v_{1},\;v_{2},\ldots ,v_{l}\right\}$, we aimed to train a model and predict the probability of these vertices forming a hyperedge.

(4) $$P(v_{1},\;v_{2},\ldots ,v_{l})=\left\{\begin{array}{c} \geq t,\;\;\;\;\;\;\;\;\;(v_{1},v_{2},\ldots ,v_{l})\in E\\ <t,\;\;\;\;\;\;\;\;\;(v_{1},v_{2},\ldots ,v_{l})\notin E \end{array}\right.$$

where t, the threshold for binarization, is typically chosen as 0.5 to binarize the continuous probability score into a label, indicating whether these vertices can form a hyperedge.

### 2.4. Hypergraph Construction

**Figure 1:**
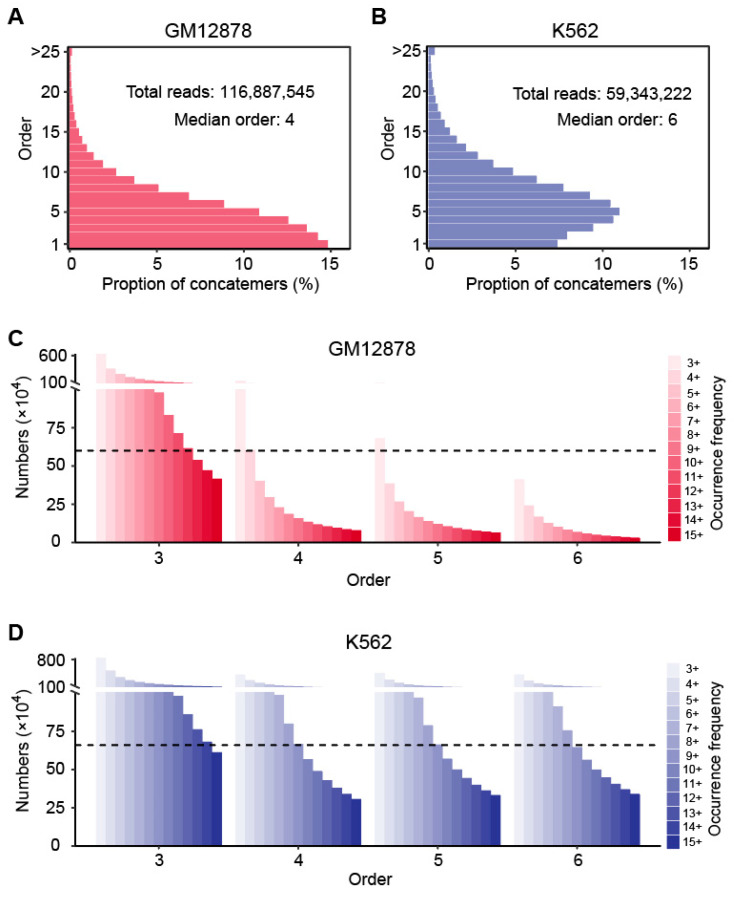
HiPore-C datasets overview and hyperedge construction. (**A**) Distribution of the contact order in the GM12878 HiPore-C concatemers. (**B**) Distribution of the contact order in the K562 HiPore-C concatemers. (**C**) Hyperedges with different occurrence frequencies constructed from GM12878 HiPore-C concatemers. The dotted line means that the retained hyperedges were used in the EpiMCI. The hyperedge numbers of different orders were kept as consistent as possible. (**D**) Hyperedges with different occurrence frequencies constructed from the K562 HiPore-C concatemers.

The hypergraph was constructed based on GM12878 and K562 HiPore-C multi-way chromatin interaction data [21]. Specifically, we divided the whole genome into continuous 1 Mb non-overlapping bins and assigned a bin index to the fragment if the fragment was located within the bin. Then, we regarded it as a hyperedge when multiple fragments belonged to the same read. Note that we select the hyperedges with the order of three to six due to their abundance among all the hyperedges (Figure 1). We also counted the occurrence frequency of each hyperedge and selected a suitable frequency threshold to maintain the balance among the hyperedges with different orders. Considering our classification task, we performed a negative sample construction. Here, we treated the filtered hyperedges mentioned above as the positive samples. Inspired by Zhang et al. [37], we generated two times the amount of negative samples by randomly sampling some vertices in the positive samples and replacing these vertices with others. The sampling strategy adopted a zero truncated binomial distribution and the replacement maintained a similarity between the positive and negative samples so that they could not be distinguished simply by simple metrics, such as the 1D genomic distance, which ensured the preciseness and correctness of the experiment.

### 2.5. Vertex Feature Generation

We used functional genomic signals on the 1 Mb genomic bins (histone mark ChIP-seq, TF ChIP-seq and ATAC-seq of both GM12878 and K562 cell lines, https://www/encodeproject.org accessed on 14 March 2023) as the vertex features. In this way, we could improve the generalization ability of the model, which could accurately predict multi-way interactions by solely using 1D genomic signals instead of depending on the hypergraph itself adopted by the previous study. In addition, we made full use of the available epigenetic data for our prediction and mitigated considerable trouble in the wet experiment, which can be time consuming and expensive.

**Figure 2:**
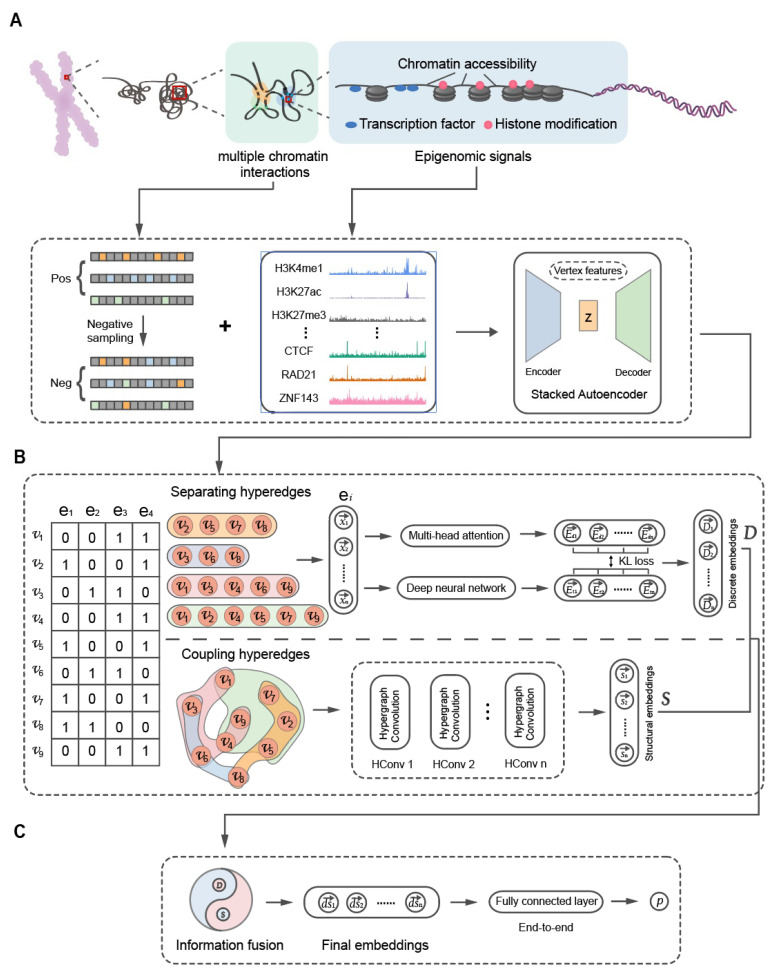
Overview of the EpiMCI framework. (**A**) The pos/neg samples and vertex features generation. The epigenomic signals on the 1 Mb genomic bins were taken as the input of the SAE and converted into 64-dimension feature embeddings. (**B**) The EpiMCI consisted of two modules: separating hyperedges representation learning (top) and coupling hyperedges representation learning (bottom). The separating hyperedges representation learning fed the feature embeddings into a multi-head self-attention layer and a DNN layer to obtain the vertex discrete embeddings. The coupling hyperedges representation learning used the feature embeddings as the input for the hypergraph convolution operation to obtain the vertex structural embeddings. (**C**) The final vertex embeddings were obtained after the feature fusion between the discrete embeddings and structural embeddings and passed through a fully connected layer to predict the final probability score.

There were several proteins that tended to function together in our original features. For example, the CTCF, SMC and RAD21 proteins worked together to facilitate the formation of a 3D genome organization, which introduced feature redundancy and noise (i.e., insignificant features) [40,41]. Therefore, we introduced a stacked autoencoder (SAE), composed of multi-layer autoencoders (AEs), to denoise and transform the data from a higher-dimensional space to a lower-dimensional feature space (Figure 2A) [42,43]. An AE is a forward neural network consisting of an input layer, a hidden layer and an output layer. It’s designed to obtain new attributes from hidden layer outputs. The hidden layer can be described as follows.

(5) $$y_{i}=\sigma (W^{T}x_{i}+b)$$

where W is the weight matrix of the encoder, b is the bias value and σ is the activation function of the encoder, chosen as $\sigma =ReLU\left(\cdot \right)$. The output z can be obtained as follows.

(6) $$z_{i}=\sigma ({W^{’}}^{T}x_{i}+b’)$$

where $W^{\prime }$ is the weight matrix of the decoder, b′ is the bias value and σ is the activation function of the decoder, chosen as $\sigma =ReLU\left(\cdot \right)$. Then we aimed to minimize the objective loss function to train the AE model.

(7) $$L_{recon}(X,\;Z)=\frac{1}{N}\sum_{i=1}^{N}{\left||x_{I}\;–\;z_{i}|\right|}^{2}$$

The SAE was similar to the AE. Specifically, the hidden layer vector of the upper layer was taken as the input of the next layer of the autoencoder. The last hidden layer vector was used as the final feature embedding vector [44]. Here, we used two hidden layers and set the hidden layers to 128. Since we used the epigenomic signals of each vertex as its feature, the prediction function can be rewritten as follows.

(8) $$P(v_{1},\;v_{2},\ldots ,v_{l})=P(f(x_{1}),\;f(x_{2}),\ldots ,f(x_{l}))$$

where $f(x_{i})$ is the embedding vectors from high-dimensional features for the vertex $v_{i}$.

### 2.6. EpiMCI Model Architecture

We proposed the EpiMCI, a novel hypergraph representation learning approach based on dual-channel hypergraph neural networks, to learn the vertex embeddings. The framework of the EpiMCI is depicted in Figure 2 and described as follows. Given a hypergraph, we obtained the vertex embedding vectors from two perspectives: separating hyperedges and coupling hyperedges (Figure 2B). The former only considered the information of a single hyperedge and used each hyperedge as the input for model training, while the latter mainly focused on the relation among the different hyperedges (detailed below). The joint representation learning assured the representation complementarity from the separating hyperedges and coupling hyperedges. After obtaining the discrete embedding and structural embedding from the separating hyperedges and coupling hyperedges, respectively, we applied an integration strategy based on a neural network to perform the feature fusion. The final embedding was used to calculate the probability score p in an end-to-end manner, which passed the embedding vector into a fully connected layer to determine whether these vertices could form a real hyperedge.

### 2.7. Vertex Representation from Separating Hyperedges

We denoted the input by n-length tuples, i.e.,$\;X=(\vec{x_{1}},\vec{x_{2}},\ldots ,\vec{x_{n}})$. Firstly, the n-length tuples were separately taken as the input of a multi-head self-attention layer and transformed into the corresponding vertex embedding vectors called dependent embeddings due to their dependence on the other vertices within the same tuple [45]. The multi-head self-attention mechanism of the transformer blocks helped select the important features and endowed them with higher weights for accurate precision [46]. The scale-dot product attention required three inputs: Q(query), K(key), V(value), which were the linear transformation of input *X*.

(9) $$Q=X\times W_{Q}\;$$

(10) $$K=X\times W_{K}$$

(11) $$V=X\times W_{V}$$

where $W_{Q}$, $W_{K}$ and $W_{V}$ are the learnable weight matrices.

Given the three learnable weight matrices, $W_{Q}$, $W_{K}$ and $W_{V}$, the attention score indicating the pairwise importance of the vertices could be calculated as follows.

(12) $$e_{ij}={\left(W_{Q}^{T}x_{i}\right)}^{T}\left(W_{K}^{T}x_{j}\right),\;\;\forall 1\leq i,j\leq n$$

It can be further normalized by the softmax function.

(13) $$\alpha_{ij}=softmax(e_{ij})=\frac{exp(e_{ij})}{\sum_{1\leq p\leq n}exp(e_{ip})}$$

The final vertex embeddings encoded using multi-head attention, denoted by $\vec{E_{d}}$, could be computed using a non-linear activation function.

(14) $$E_{di}=\sigma (\sum_{1\leq j\leq n,\;j\neq i}{\alpha_{ij}W}_{V}^{T}x_{j})$$

where we selected $\sigma \left(\cdot \right)=tanh\left(\cdot \right)$ as the activation function.

The n-length tuples also passed through a deep neural network (DNN) to obtain the embedding vectors $\vec{E_{I}}$, called independent embeddings due to its independence from the other vertices within the same tuple.

(15) $$E_{li}=W_{3}\sigma (W_{2}\sigma (W_{1}x+b_{1})+b_{2})+b_{3}$$

where σ represents the activation function ReLu, $W_{1}$, $W_{2}$ and $W_{3}$ are the learnable weight matrices, respectively, and $b_{1}$, $b_{2}$ and $b_{3}$ are the bias vectors for the corresponding layers of the MLP.

To capture the consistent representation between $\vec{E_{d}}$ and $\vec{E_{i}}$, we introduced the Kullback–Leibler (KL) divergence loss to perform joint optimization and identify a common subspace. Thus, we obtained the resulting joint representations, also called discrete embeddings $\vec{D}$, from the separating hyperedges.

(16) $$D_{KL}(P_{ED},\;P_{EL})=\sum_{k}P_{ED}(x_{i}){log}_{2}\frac{P_{ED}(x_{i})}{P_{EL}(x_{i})}$$

where $P_{ED}$ and $P_{EL}$ represent the distribution of the different separating hyperedge representation maps.

### 2.8. Vertex Representation from Coupling Hyperedges

In this perspective, we mainly focused on the common vertices shared by the hyperedges. We exploited a hypergraph convolution operation (Hconv) to identify the discriminative vertex embeddings [47].

(17) $$X^{\left(t+1\right)}=\sigma (D_{v}^{-1/2}HWD_{e}^{-1}H^{T}D_{v}^{-1/2}X^{\left(t\right)}W_{t})$$

where $X^{\left(t\right)}$ and $X^{\left(t+1\right)}$ are the input of the (*t*)th and (*t* + 1)th layer, respectively; $D_{v}$, $D_{e}$ are the diagonal matrices of the vertex and hyperedge degrees, respectively; H is the hypergraph incidence matrix; W is the diagonal matrix of the hyperedge weights; $W_{t}$ is the weight matrix between the (*t*)th and (*t* + 1)th layer that can be identified during the training process and $\Sigma \left(\cdot \right)$ is a non-linear activation function. We choose LeakyReLU here [48] and we adopted three convolutional layers. After convolution, we could obtain the final refined structural embeddings S.

Finally, we generated the fusion embeddings by feeding the discrete embeddings and structural embeddings into a dense layer (Figure 2C). It could be formulated as follows.

(18) $$DS=f\left(D\;⨁S\right)$$

where ⨁ is the concatenation and fusion operator, $f\left(\cdot \right)$ is the dense layer projection operation and DS is the final vertex fusion embedding.

It was then further passed through a fully connected layer with sigmoid as the activation function [49], which is widely used to address binary classification tasks [50], to produce a probability score $p_{i}$. Finally, all the outputs $p_{i}\;\in [0,1]$ were averaged to obtain the final result p.

(19) $$p=\frac{1}{l}\sum_{i=1}^{l}p_{i}=\frac{1}{l}\sum_{i=1}^{l}\sigma ({DS}_{i})$$

(20) $$\sigma \left(x\right)=\frac{1}{\left(1+e^{-x}\right)}$$

To measure the error between the predicted and real labels, the cross-entropy loss function could be defined as [51].

(21) $$L=-\frac{1}{N}\sum y\log\hat{y}+(1-y)\log\left(1-\hat{y}\right)$$

Hence, an end-to-end model architecture was constructed.

### 2.9. Experiment Setting and Evaluation Metrics

The EpiMCI was implemented in Python based on pytorch (version 1.12.0). The embedding dimension was set to 64. The learning rate of the Adam optimizer was set to 1 × 10^−3^ and the training epochs were set to 200. All the experiments were conducted using a 32-core machine with a NVIDIA GeForce RTX 3090 GPU card.

In this experiment, a five-fold cross-validation (5-CV) was used to evaluate the performance of the EpiMCI. We also adopted several classification metrics to measure the prediction performance, including the area under the receiver operating characteristic curve (AUC), the area under the precision-recall curve (AUPR), accuracy, precision, recall and F1 score (F1).

## 3. Results and Discussion

### 3.1. Hyperedge Generation

The recently released GM12878 and K562 HiPore-C data were used to construct the hyperedges. As described in the previous study, they greatly increased the output of multi-way contact sequencing by solving nanopore clogging, which limited the throughput of the traditional Pore-C method [21]. Taking the data quality into account, we adopted the HiPore-C data and achieved ~116M and 67M valid multi-contact reads for GM12878 and K562, respectively (Figure 1A,B). The multiple fragments covered by a read were regarded as the vertices under a 1 Mb resolution, which further connected each read into a hyperedge. To improve the data accessibility, we decomposed the higher-order contacts into several lower-order hyperedges (order ≥ 3). The number of generated hyperedges is listed in Table S1. We selected different occurrence frequency thresholds to maintain the sample size balance among the hyperedges that had various orders (Figure 1C,D). Specifically, for GM12878, we set the frequency threshold to 12 with the order of three, four with the order of four, three with the order of five and three with the order of six, resulting in 615,731, 604,243, 680,788 and 412,131 hyperedges corresponding to the orders of three, four, five and six, respectively. For K562, we set frequency threshold to 14 with the order of three, nine with the order of four, nine with the order of five and nine with the order of six, which resulted in 681,028, 665,728, 662,930 and 645,731 hyperedges corresponding to the orders of three, four, five and six, respectively.

### 3.2. Multi-Way Chromatin Interaction Prediction

The EpiMCI is a framework based on hypergraph neural networks to predict underlying multi-way chromatin interactions (Figure 2). Here, we regarded the multi-way chromatin interaction prediction problem as a classification task, i.e., given a group of vertices, we needed to determine whether they could form a hyperedge. Therefore, we used a total of six model classification metrics to evaluate the prediction performance of the EpiMCI comprehensively, including the area under the receiver operating characteristic curve (AUC), the area under the precision-recall curve (AUPR), accuracy, precision, recall and F1 score. The performance is detailed in Table S2 and Figure 2. A resolution of 1 Mb was used to explore the multi-way chromatin interactions, as well as MATCHA, which was proposed by Zhang et al. in 2020 [37].

### 3.3. Model Performance Comparison

**Figure 3:**
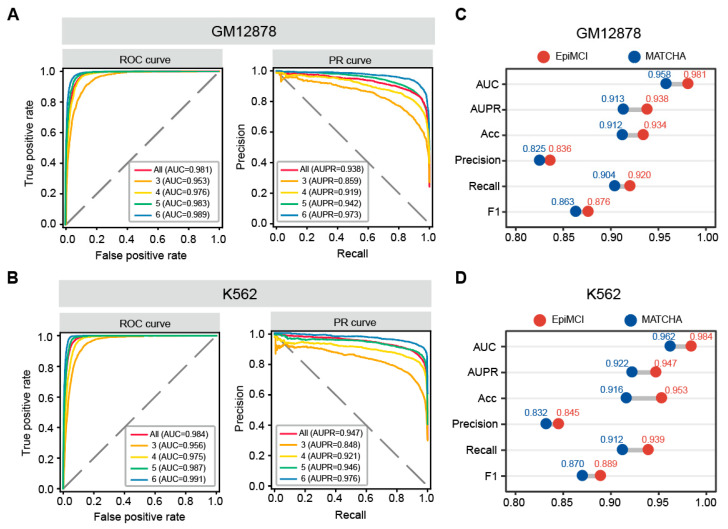
Overall performance of the EpiMCI and comparison with MATCHA. (**A**) ROC curve and PR curve of the EpiMCI among different orders for GM12878. “All” means it was evaluated using hyperedges merged from all the orders. (**B**) ROC curve and PR curve of the EpiMCI for K562. (**C**) Performance comparison with MATCHA for GM12878. The six classification evaluation metrics are listed. (**D**) Performance comparison with MATCHA for K562. The red circles denote the EpiMCI, and the blue circles denote MATCHA.

We trained and evaluated the EpiMCI in terms of six evaluation metrics. We also listed the evaluation performance metrics of MATCHA, which was used to perform a comparison with our model (Table S2). Undoubtedly, MATCHA achieved a good performance with an overall AUC of 0.958 and an average AUPR of 0.913 for GM12878 (Figure 3C). Indeed, Zhang et al. developed MATCHA using GM12878, SPRITE and Drosophila S2 RNAPII ChIA-Drop data [37]. They achieved AUC values ranging from 0.845 to 0.987 for contact orders from five to three for GM12878, where the lower AUC score seemed to be caused by the lower occurrence frequency. This was partly indicative of the efficiency of the HiPore-C data, which was optimized from the Pore-C protocol and had a more substantial increase in the output compared to the other methods.

Compared to MATCHA, the EpiMCI showed a better performance with an average AUC score of 0.981 and an AUPR score of 0.938 using the GM12878 data (Figure 3A). For the K562 data, the AUC score was 0.984 and the AUPR score was 0.947 (Figure 3B). The EpiMCI was able to make accurate predictions across the hyperedges with different orders in both GM12878 and K562 (Figure 3C,D). It is worth noting that the classification performance increased with the number of hyperedge orders. In this study, the hyperedges mainly consisted of two parts. One was the fragment numbers observed by the original sequencing data and the other was the sources from the decomposition of the hyperedges with higher orders. The hyperedge construction could have accounted for the result above since we kept the hyperedge numbers consistent among the different orders, which resulted in redundant vertices among the hyperedges within small orders, such as three and four. The redundant information did not predict the performance as well as the hyperedges with large order and non-redundant vertices. Overall, this evaluation suggested that the EpiMCI was able to accurately predict a fraction of the hyperedges derived from multi-way chromatin interaction data along with their epigenetic information.

### 3.4. Ablation Experiment

**Figure 4:**
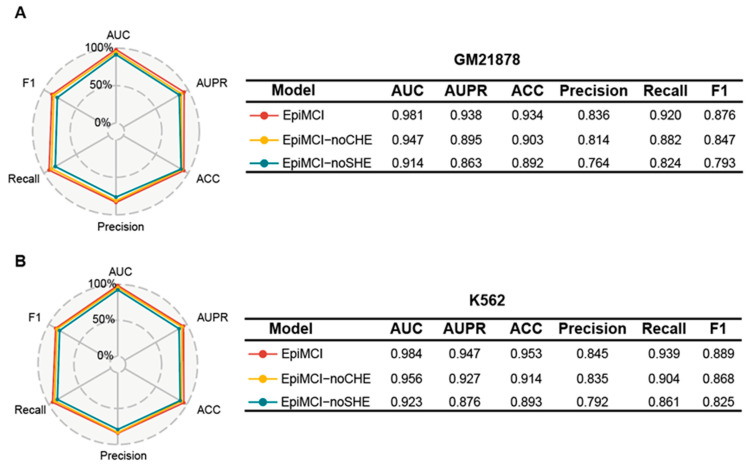
Experiment results of the ablation analysis. (**A**) Ablation results for GM12878. (**B**) Ablation results for K562. The EpiMCI-noCHE means that we removed the coupling hyperedges representation of the EpiMCI, and the EpiMCI-noSHE means that the separating hyperedges representation was omitted.

To measure the power of the different components in the EpiMCI, we conducted an ablation analysis. Specifically, we constructed two variants of the EpiMCI, named the EpiMCI-noSHE and EpiMCI-noCHE. The EpiMCI-noSHE removed the separating hyperedges representation, whereas the EpiMCI-noCHE skipped the coupling hyperedges information. With a five-fold cross validation of each method, we compared their performance to the EpiMCI in both the GM12878 and K562 datasets. In GM12878, the mean AUC values for the EpiMCI, the EpiMCI-noSHE and EpiMCI-noCHE were 0.981, 0.947 and 0.914, respectively, and the AUPR values were 0.938, 0.895 and 0.863, respectively (Figure 4A). For the K562 data, the mean AUC values for the EpiMCI, the EpiMCI-noSHE and EpiMCI-noCHE were 0.984, 0.956 and 0.923, respectively, and the AUPR values were 0.947, 0.927 and 0.876, respectively (Figure 4B). In addition, the ACC, precision, recall and F1 score of the EpiMCI all consistently surpassed the other methods in both datasets. The large decrease in the evaluation metrics in the EpiMCI-noSHE proved that the information underlying the separating hyperedges is important for prediction and the coupling hyperedges are integral for boosting the performance of the EpiMCI.

### 3.5. Optimization of Model Hyperparameters

**Figure 5:**
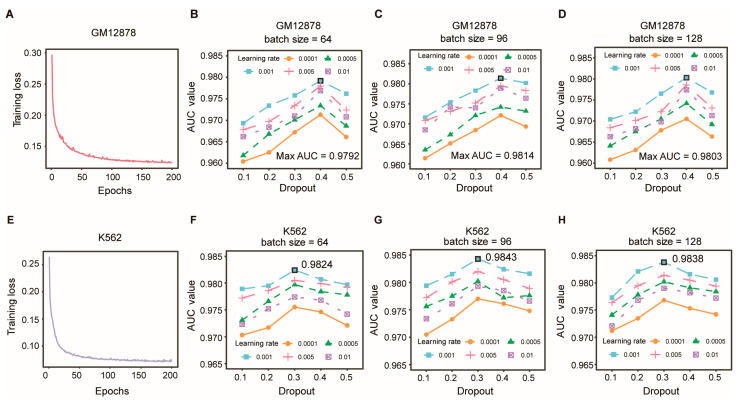
Hyperparameter sensitivity analysis of the GM12878 and K562 HiPore-C datasets. (**A**) Training loss change with the epochs during model training for GM12878. (**B**–**D**) The impact of the different hyperparameters on the model performance when the batch size was 64 (**B**), 96 (**C**) and 128 (**D**). (**E**) Training loss change with the epochs during model training for K562. (**B**–**D**) The impact of the different hyperparameters on the model performance when the batch size was 64 (**F**), 96 (**G**) and 128 (**H**).

In this section, we first explored the model convergence by observing the change in the training loss with the epochs during the training process. As shown in Figure 5A, the loss training in the GM12878 datasets converged at the 200th epoch, which implied a reasonability and acceptability of the model. It was the same when training on the K562 datasets (Figure 5E). In addition, the deep learning models could perform differently with the different hyperparameter values. To illuminate how the hyperparameters impact the predictive performance of the proposed model, we applied a grid search and a five-fold CV on the GM12878 HiPore-C dataset to obtain the optimal values of the following hyperparameters: the batch size (s), dropout (d) and learning rate (lr). The s was selected from {64, 96, 128}, d was selected from {0.1, 0.2, 0.3, 0.4, 0.5} and lr was selected from {0.0001, 0.0005, 0.001, 0.005, 0.01}.

The experiment results suggested that the EpiMCI achieved the best performance in GM12878 when s was 96, d was 0.4 and lr was 0.001, which were selected in the following sections. As illustrated in Figure 5B–D, when s increased from 64 to 128, the performance of the model increased and achieved the best performance at s = 96, after which the performance declined. The results indicated that the batch size in the model training could generally impact the final model performance. Additionally, the model performance improved with increasing dropout, reaching its optimum at d = 0.4, and then began to decline. The model clearly outperformed its counterparts when lr = 0.001. This suggested that the hyperparameter selection was essential in model training. The values away from the optimum caused performance degradation. We also explored the optimal hyperparameters (s = 96, d = 0.3, lr = 0.001) for the K562 HiPore-C dataset and the results are detailed in Figure 5F–H.

### 3.6. Case Studies

#### 3.6.1. EpiMCI Improves HiPore-C Data Quality

**Figure 6:**
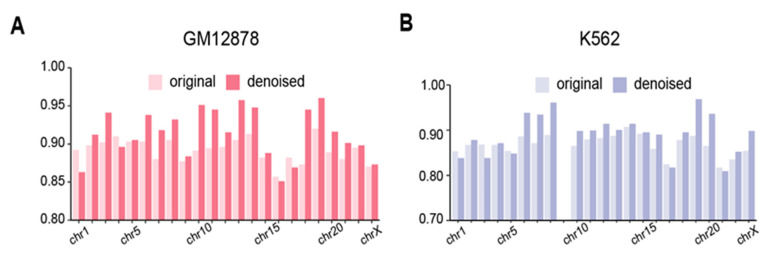
Denoising performance of the EpiMCI. (**A**) GenomeDISCO score for each chromosome of Hi-C versus HiPore-C (original) and the EpiMCI denoised HiPore-C (denoised) in GM12878. (**B**) GenomeDISCO score for each chromosome of Hi-C versus HiPore-C (original) and the EpiMCI denoised HiPore-C (denoised) in K562.

To further access the performance of the EpiMCI, we applied it to denoise the whole-genome chromatin interaction, as the noise introduced by experiments, such as randomly ligated DNA, was inevitable for the technique [52,53]. Here, we obtained all the pairs of 1 Mb genomic bins and used them as the input for the trained model. The resulting probabilities of pairwise interactions was reshaped as an N × N matrix and denoted as a likelihood matrix. Then, the denoised contact matrix was calculated using the element-wise product of the likelihood matrix and the original contact matrix, which was defined as the HiPore-C contact matrix decomposed from a chromatin multi-way interaction. We compared the denoised contact matrix to the original contact matrix and Hi-C contact matrix using the GenomeDISCO score, a concordance measurement proposed to assess the similarity of a pair of contact maps obtained from chromosome conformation capture experiments [39]. The denoised map improved as a whole in both GM12878 and K562, although there were several exceptions such as in chr1, chr16 and chr17 (Figure 6A,B). Note that chromosome 9 in the K562 data was excluded due to its high sparsity, which was also mentioned in a previous study [54]. Overall, the results showed that the EpiMCI could be applied to improve the HiPore-C data and has potential to be generalized to other types of data for capturing chromatin multi-way interaction, such as the SPRITE, ChIA-Drop and Pore-C datasets.

#### 3.6.2. EpiMCI Reflects 3D Genome Global Positioning Patterns

**Figure 7:**
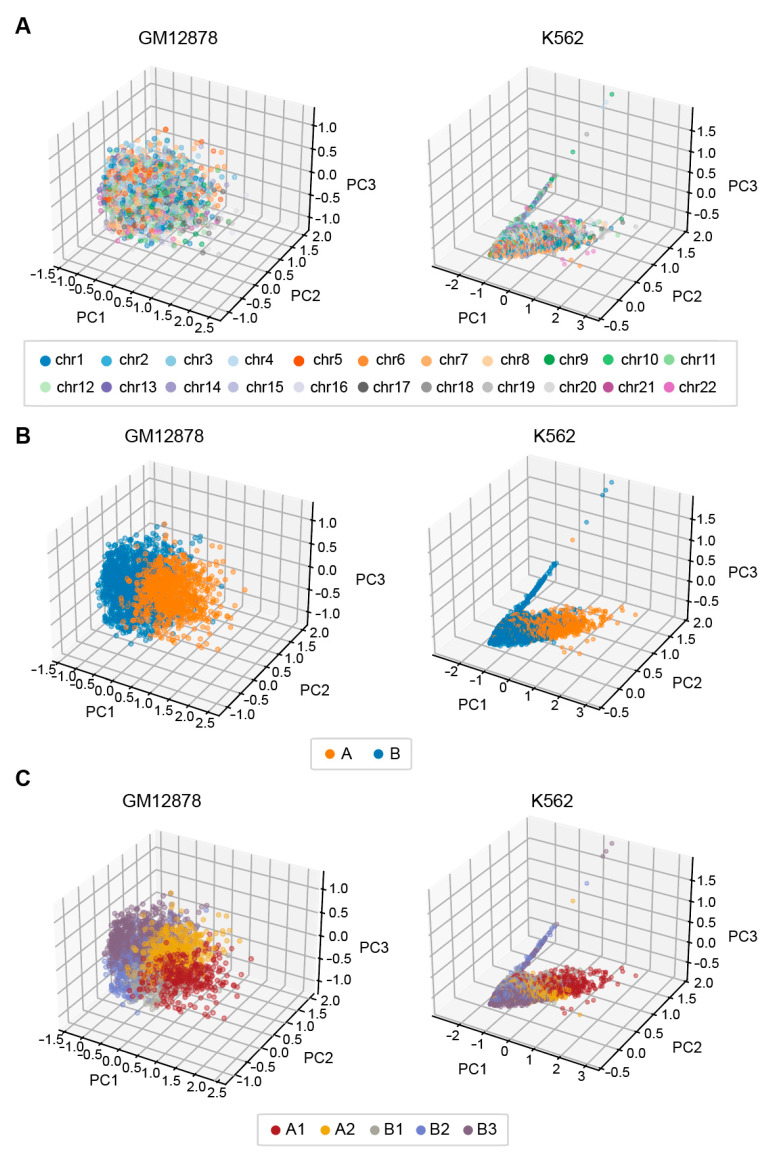
Vertex embeddings extracted from the EpiMCI reflecting the 3D genome organization. (**A**) Embedding visualization of GM12878 (**left**) and K562 (**right**). The embeddings were processed using a PCA and the points (bins) were colored based on the chromosomes they belong to. (**B**) Embedding visualization of GM12878 (**left**) and K562 (**right**). The embeddings were processed using a PCA and the points (bins) were colored based on the A/B compartments they belong to. (**C**) Embeddings visualization of GM12878 (**left**) and K562 (**right**). The embeddings were processed using a PCA and the points (bins) were colored based on the sub-compartments they belong to.

To explore whether the EpiMCI could obtain biologically meaningful information from plenty of multi-way interactions, we extracted the embeddings generated by the EpiMCI trained from GM12878 and K562. We used 1 Mb genomic bins for this analysis. As is widely known, chromosome decondensation leads to the formation of nuclear territories, which are further spatially segregated into two chromosome compartments predominantly consisting of either euchromatic (the A compartment) or heterochromatic (the B compartment) genome segments [6,55]. In the 1 MB resolution contact matrix heatmap, we observed an obvious chessboard pattern which consisted of both A and B compartments. Several studies also provided evidence for sub-compartments and demonstrated that these sub-compartments exhibited distinct genomic and epigenomic contents [7,56,57]. Under this background, we extracted the embeddings generated by the EpiMCI and performed a principal component analysis (PCA) using the scikit-learn package for visualization. We firstly checked if there were preferences since intra-chromosomal contacts tend to predominate in a chromatin capture experiment. The results revealed a scattered distribution of genomic bin locations, although some bins belonged to the same chromosome, which demonstrated that our method processing multi-way interaction data was unbiased (Figure 7A). Then, we performed the same analysis against the compartments and sub-compartments. For the compartments, the genomic bins were separated clearly along the PC1, indicating that the model captured the intrinsic features of the data (Figure 7B). As for the sub-compartments, the bins seemed to be divided into five groups as a whole. They were arranged as B3, B2, B1, A2 and A1 from left to right along the PC1, corresponding to the exact activities of the genomic regions (Figure 7C). The bins located around the A/B compartment demarcation line were mixed together, especially the bins within the A2 and B3 sub-compartments. The fact that these bins corresponded to a relatively weak active or inactive degree rendered difficulty in partitioning them into distinct clusters.

## 4. Conclusions

In this paper, we proposed a model based on hypergraph representation learning, named EpiMCI, to predict 3D chromatin multi-way interactions. It used epigenomic information as the input and extracted key features for prediction. We compared our model with the baseline model, MATCHA, and showed that the EpiMCI had a better performance than MATCHA. Different from MATCHA, the EpiMCI introduced not only separating hyperedges, but also coupling hyperedges, which focused on shared vertices among the different hyperedges, thus generating a more comprehensive feature representation and achieving improved performances. The shared vertices tended to be chromatin interaction connection hubs and occupied a more important position than others. We also confirmed this inference using ablation experiments. In addition, the EpiMCI has further biological applications. Firstly, it could be applied to remove noise introduced by experiments, improving the signal–noise ratio of the HiPore-C datasets. It also has the potential to be generalized to diverse datasets, such as SPRITE, ChIA-Drop, etc.. Second, the resultant embeddings reflected a 3D genome organization at the compartment level. Although the resolution we selected, i.e., 1 Mb, limited a more detailed exploration of the biological meaning, our result demonstrated its significance in reflecting 3D spatial positioning and uncovering genuine chromatin conformation. Last, the EpiMCI could reconstruct multi-way chromatin contact maps using epigenomic signals, facilitating studies that aim to explore chromatin interactions with only the epigenome, thereby mitigating expensive sequencing costs.

Despite the fact that the EpiMCI can accurately predict the multi-way chromatin interactions solely based on epigenomic data, there is still room for improvement. As mentioned above, low resolution leads to limitations for investigation in greater detail, such as TAD level and loop level. A fine-grained analysis is required to promote fine-scale 3D organization. We only implemented experiments using the available HiPore-C data. The generalization ability of the EpiMCI has not been explored in other similar datasets. Furthermore, the basic sequence information can be introduced to improve the performance of multi-way chromatin interaction prediction. For chromatin regions which are transcription poor and recombination prone, such as the centromere and telomere regions, the predictive ability of the EpiMCI may be greatly discounted due to its dependence on epigenomic information. In addition, due to the limited epigenomic information and insufficient experimental conditions, we have not verified the model performance on different chromatin structures, such as bacterial chromatin in log versus stationary phase cultures or in eukaryotic cells chromatin status in G1 phase versus mitotic phase of the cell cycle. It is challenging for us to improve the model so that the EpiMCI could adapt to different scenarios and handle multiple tasks. Further exploration by simultaneously considering the multiple factors mentioned above will be widely applicable to mining multi-way chromatin interactions within the genome and can help solve more complex biological problems.

## Data Availability

The preprocessed data will be made available on request.

## Supplementary Materials

The following supporting information can be downloaded at: https://www.mdpi.com/article/10.3390/biology12091203/s1. Table S1. Hyperedges in the GM12878/K562 HiPore-C datasets. Table S2. EpiMCI performance and comparison with MATCHA. Table S3. Epigenomic signals used for EpiMCI prediction.
