# Supplementary material for: EpiMCI: Predicting Multi-Way Chromatin Interactions from Epigenomic Signals
Source: Biology (Basel). 2023 Sep 3;12(9):1203. doi: 10.3390/biology12091203 (PMC10525350; doi:10.3390/biology12091203)
Supplement: Supplementary file 1 [file biology-12-01203-s001.zip › biology-2554204-supplementary.pdf]

EpiMCI: Predicting Multi-Way Chromatin Interactions from Epigenomic Signals

Jinsheng Xu [1,†], Ping Zhang [1,†], Weicheng Sun [1], Junying Zhang [1], Wenxue Zhang [2], Chunhui Hou [3,*] and Li Li [1,4,*]

[1] Hubei Key Laboratory of Agricultural Bioinformatics, College of Informatics, Huazhong Agricultural University, Wuhan 430070, China

[2] Food Science Program, Division of Food, Nutrition and Exercise Sciences, University of Missouri, 1406 E Rollins Street, Columbia, MO 65211, USA

[3] China State Key Laboratory of Genetic Resources and Evolution, Kunming Institute of Zoology, Chinese Academy of Sciences, Kunming 650223, China

[4] Hubei Hongshan Laboratory, Huazhong Agricultural University, Wuhan 430074, China

* Correspondence: houchunhui@mail.kiz.ac.cn (C.H.); li.li@mail.hzau.edu.cn (L.L.)

† These authors contributed equally to this work.

Table S1. Hyperedges in GM12878/K562 HiPore-C datasets.

	GM12878				K562			
**Freq**	**3**	**4**	**5**	**6**	**3**	**4**	**5**	**6**
**2+**	25,499,786	4,732,929	3,892,251	3,793,817	45,000,094	12,001,359	15,590,988	20,780,406
**3+**	6,627,056	1,086,686	**680,758**	**412,131**	10,776,737	3,915,725	4,314,851	3,916,687
**4+**	3,440,588	**604,243**	385,150	241,428	5,012,706	2,400,526	2,484,406	2,226,432
**5+**	2,358,800	402,027	266,881	168,281	3,221,949	1,675,365	1,676,680	1518,271
**6+**	1,794,906	294,301	203,797	126,908	2,384,748	1,255,113	1,237,340	1,140,987
**7+**	1,431,160	228,960	165,148	100,397	1,889,167	984,958	967,584	910,446
**8+**	1,173,726	186,196	139,070	82,053	1,554,394	799,234	788,817	755,723
**9+**	980,180	156,352	120,021	68,503	1,310,852	**665,728**	**662,930**	**645,731**
**10+**	831,098	134,635	105,349	58,104	1,125,334	566,031	570,192	562,820
**11+**	712,168	118,236	93,786	49,977	978,874	489,797	499,482	498,795
**12+**	**615,731**	105,116	84,354	43,619	860,682	429,107	443,789	447,641
**13+**	537,270	94,756	76,596	38,490	762,378	379,914	399,338	405,477
**14+**	471,313	86,356	70,065	34,263	**681,028**	340,137	362,757	370,379
**15+**	416,070	79,252	64,493	30,755	612,156	306,697	332,438	340,312

Table S2. EpiMCI performance and comparison with MATCHA.

Cell line	Model	Order	AUC	AUPR	Acc	Precision	Recall	F1
GM12878	EpiMCI	All	0.981	0.938	0.934	0.836	0.920	0.876
		3	0.953	0.859	0.873	0.682	0.917	0.782
		4	0.976	0.919	0.931	0.841	0.894	0.867
		5	0.983	0.942	0.957	0.916	0.911	0.913
		6	0.989	0.973	0.978	0.956	0.957	0.956
	MATCHA	All	0.958	0.913	0.912	0.825	0.904	0.863
		3	0.930	0.813	0.845	0.649	0.872	0.744
		4	0.945	0.902	0.914	0.816	0.865	0.840
		5	0.958	0.931	0.936	0.895	0.891	0.893
		6	0.961	0.952	0.954	0.932	0.924	0.928
K562	EpiMCI	All	0.984	0.947	0.953	0.845	0.939	0.889
		3	0.956	0.848	0.892	0.751	0.868	0.805
		4	0.975	0.921	0.943	0.862	0.904	0.882
		5	0.987	0.946	0.961	0.925	0.918	0.921
		6	0.991	0.976	0.983	0.963	0.961	0.962
	MATCHA	All	0.962	0.922	0.916	0.832	0.912	0.870
		3	0.934	0.834	0.858	0.734	0.862	0.793
		4	0.951	0.916	0.920	0.828	0.856	0.842
		5	0.963	0.937	0.939	0.903	0.896	0.899
		6	0.968	0.956	0.957	0.938	0.927	0.932

Table S3. Epigenomic signals used for EpiMCI prediction.

Cell lines	Feature numbers	Proteins
GM12878	132	ARID3A, ARNT, ASH2L, ATF2, BACH1, BATF, BCL11A, BCL3, BHLHE40, BMI1, BRCA1, CBFB, CBX5, CEBPB, CEBPZ, CHD1, CHD2, CHD4, CREM, CTCF, CTCF, CUX1, DHS, DPF2, E2F4, E2F8, E4F1, EBF1, EED, ELF1, ELK1, EP300, EP300, ESRRA, ETS1, ETV6, EZH2, FOXK2, GABPA, GATAD2B, HCFC1, HDAC2, HDGF, IKZF1, IKZF2, IRF4, IRF5, JUNB, JUND, KDM1A, KLF5, MAFK, MAX, MAZ, MEF2A, MEF2B, MEF2C, MLLT1, MTA2, MTA3, MXI1, MYB, NBN, NFATC1, NFIC, NFXL1, NFYA, NFYB, NKRF, NR2C1, NR2C2, NR2F1, NRF1, PAX5, PAX5, PBX3, PKNOX1, POLR2A, POLR2AphosphoS2, POLR2AphosphoS5, RAD21, RBBP5, RCOR1, RELB, REST, RFX5, RUNX3, RXRA, SIN3A, SIX5, SKIL, SMAD1, SMAD5, SMC3, SPI1, SRF, STAT3, STAT5A, TAF1, TBL1XR1, TBP, TBX21, TCF12, TCF7, UBTF, USF1, USF2, WRNIP1, YBX1, YY1, ZBED1, ZBTB40, ZEB1, ZFP36, ZNF143, ZNF217, ZNF24, ZNF384, ZNF592, ZNF622, ZNF687, ZSCAN29, ZZZ3, ATAC-seq, CTCF, H2AFZ, H3K27ac, H3K27me3, H3K36me3, H3K4me1, H3K4me2,

		H3K4me3, H3K79me2, H3K9ac, H3K9me3, H4K20me1, POLR2A, RAD21, SMC3
K562	306	AGO1, ARHGAP35, ARID1B, ARID2, ARID3A, ASH1L, ATF1, ATF2, ATF3, ATF7, BACH1, BCLAF1, BCOR, BDP1, BHLHE40, BMI1, BRCA1, BRD4, BRD9, BRF1, BRF2, C11orf30, CBFA2T2, CBFA2T3, CBX1, CBX2, CBX3, CBX5, CBX8, CC2D1A, CCAR2, CCNT2, CDC5L, CEBPB, CEBPD, CEBPZ, CHAMP1, CHD1, CHD2, CHD4, CHD7, COPS2, CREB1, CREB3L1, CREBBP, CREM, CSDE1, CTBP1, CTCF, CTCFL, CUX1, DACH1, DDX20, DEAF1, DNMT1, DPF2, DROSHA, E2F1, E2F4, E2F6, E2F7, E2F8, E4F1, EGR1, EHMT2, ELF1, ELF4, ELK1, EP300, ESRRA, ETS1, ETV6, EWSR1, EZH2, FIP1L1, FOS, FOSL1, FOXA1, FOXK2, FOXM1, FUS, GABPA, GABPB1, GATA1, GATA2, GATAD2A, GATAD2B, GMEB1, GTF2B, GTF2F1, GTF3C2, HCFC1, HDAC1, HDAC2, HDAC3, HDAC6, HDGF, HES1, HLTF, HMBOX1, HMGN3, HNRNPH1, HNRNPK, HNRNPL, HNRNPLL, HNRNPUL1, IKZF1, ILF3, IRF2, JUNB, JUND, JUN, KAT2B, KAT8, KDM1A, KDM4B, KDM5B, KHSRP, KLF16, L3MBTL2, LARP7, LEF1, MAFF, MAFK, MAX, MBD2, MCM2, MCM3, MCM5, MCM7, MEF2A, MEIS2, MGA, MIER1, MITF, MLLT1, MNT, MTA1,

	MTA2, MTA3, MXI1, MYNN, NBN, NCOA1, NCOA2, NCOA4, NCOA6, NCOR1, NELFE, NEUROD1, NFATC3, NFE2, NFIC, NFRKB, NFXL1, NFYA, NFYB, NKRF, NONO, NR2C1, NR2C2, NR2F1, NR2F2, NR2F6, NR3C1, NUFIP1, PCBP1, PCBP2, PHB2, PHF20, PHF21A, PHF8, PKNOX1, PML, POLR2A, POLR2AphosphoS2, POLR2G, POLR3A, POLR3G, POU5F1, PRDM10, PRPF4, PTBP1, RAD21, RAD51, RB1, RBBP5, RBFOX2, RBM14, RBM15, RBM17, RBM22, RBM25, RBM34, RBM39, RCOR1, REST, RFX1, RFX5, RLF, RNF2, RUNX1, SAFB2, SAFB, SAP30, SETDB1, SFPQ, SIN3A, SIN3B, SIRT6, SIX5, SKIL, SMAD1, SMAD2, SMAD5, SMARCA4, SMARCA5, SMARCB1, SMARCC2, SMARCE1, SMC3, SNIP1, SNRNP70, SOX6, SP1, SP2, SPI1, SREBF1, SRF, SRSF1, SRSF3, SRSF7, SRSF9, STAT5A, SUPT5H, SUZ12, TAF15, TAF1, TAF7, TAL1, TARDBP, TBL1XR1, TBP, TCF7, TCF7L2, TEAD4, THAP1, THRA, TOE1, TRIM24, TRIM25, TRIM28, U2AF1, U2AF2, UBTF, USF1, USF2, WHSC1, XRCC3, XRCC4, XRCC5, YBX1, YY1, ZBED1, ZBTB11, ZBTB33, ZBTB5, ZBTB7A, ZBTB8A, ZC3H11A, ZC3H8, ZEB2, ZFP36, ZFP91, ZHX1, ZKSCAN1, ZMIZ1, ZMYM3, ZNF143, ZNF24, ZNF263, ZNF274, ZNF280A, ZNF282,

	ZNF316, ZNF318, ZNF384, ZNF407, ZNF592, ZNF639, ZNF830, ZSCAN29, ZZZ3, ATAC-seq, CTCF, H2AFZ, H3K27ac, H3K27me3, H3K36me3, H3K4me1, H3K4me2, H3K4me3, H3K79me2, H3K9ac, H3K9me1, H3K9me3, H4K20me1, POLR2A, RAD21, SMC3
